# Prevalence of Rheumatoid Factor and Anti-citrullinated Protein Antibodies in Patients With Post-treatment Lyme Disease

**DOI:** 10.7759/cureus.82541

**Published:** 2025-04-18

**Authors:** John B Miller, Alison Rebman, Ting Yang, John Aucott

**Affiliations:** 1 Department of Medicine, Johns Hopkins University School of Medicine, Baltimore, USA; 2 Department of Medicine, Johns Hopkins University, Baltimore, USA

**Keywords:** anti-citrullinated protein antibodies, lyme disease, post-treatment lyme disease syndrome (ptlds), rheumatoid arthritis, rheumatoid factor (rf)

## Abstract

Post-treatment Lyme disease (PTLD) occurs in a portion of patients after initial antibiotic treatment of Lyme disease (LD) and is often characterized by arthralgia without synovitis. Rheumatoid factor (RF) and anti-citrullinated protein antibodies (ACPA) are often used to assess joint pain in this setting; however, their clinical utility remains unknown. Our objective was to define the frequency of these autoantibodies in a large cohort of carefully characterized patients with PTLD meeting a research case definition and to determine the clinical implications of these tests. RF and ACPA were tested as indicated clinically and abstracted by chart review. The prevalence of antibodies and their relationship to symptoms were examined. Of the 167 patients included in the analysis, RF status was documented at least once for 78.4% (131 of 167), and ACPA status was available at least once for 88.0% (147 of 167). RF was positive in 3.8% (five of 131), and ACPA was positive in 4.8% (seven of 147) at least at one time point. A total of 7.2% (12 of 167) patients were found to have a positive RF or ACPA test at least at one time point. There was no difference in the proportion of patients with RF and/or ACPA based on the initial presenting manifestations of their LD, nor the symptoms of PTLD at later evaluation; however, the small sample size may limit our ability to detect these clinical differences. We found a low prevalence of RF and ACPA in this study, similar to the known rates in the general population. This reflects the lack of inflammatory arthritis in this population with clinically defined PTLD and arthralgia only.

## Introduction

Lyme disease (LD), caused by *Borrelia burgdorferi* (Bb), is one of the most common vector-borne illnesses in the United States, infecting an estimated 475,000 individuals each year [1]. Antibiotic therapy leads to symptom resolution for most patients; however, 10-15% of patients have persistent musculoskeletal pain, neurocognitive deficits, and/or fatigue severe enough to cause physical dysfunction, termed post-treatment LD (PTLD) [2]. The musculoskeletal pain of PTLD varies between individuals, but by definition, it is non-inflammatory and specifically without synovitis, enthesitis, or other manifestations of inflammatory arthritis. 

However, PTLD is not the only post-treatment sequelae associated with joint pain. Arvikar et al. described a case series of 30 patients who developed rheumatoid arthritis (RA), psoriatic arthritis, or spondyloarthritis after adequately treated LD [3]. These patients often had polyarticular synovitis associated with other disease-specific manifestations (e.g., rheumatoid factor [RF], psoriasis, and enthesitis) and showed improvement with the use of immunosuppression. However, the clinical relevance of this association is unclear, particularly given the number of new Lyme infections each year. In a small cohort of patients evaluated in a rheumatology clinic for persistent joint pain after treated LD, up to 18% (16 of 86) of patients were found to have RA or psoriatic arthritis [4]. While this is likely an overestimation of the frequency of autoimmune arthritis after LD, this suggests that clinicians do need to consider these diagnoses after treatment for infection with Bb.

Given the difficulty in differentiating inflammatory from non-inflammatory arthralgia, clinicians often use biomarkers to evaluate for inflammatory arthritis, including RF and anti-citrullinated protein antibodies (ACPA). However, interpreting these serologies alone may be difficult as there is a risk for false positive testing, particularly after infections where autoantibodies may develop transiently but may not relate to underlying autoimmunity [5,6]. The objective of this study was to understand the frequency and clinical implications of RF and ACPA autoantibodies in a large cohort of patients with well-defined PTLD.

## Materials and methods

Patients with PTLD enrolled in a clinical case series at the Johns Hopkins Lyme Disease Research Center (LDRC), Baltimore, MD, were included in this retrospective study [7]. To confirm LD, patients were required to have either (1) evidence of a physician-documented erythema migrans (EM) rash, (2) evidence of a disseminated Lyme manifestation (i.e., Lyme arthritis, neuroborreliosis, and carditis) with laboratory evidence of infection following CDC recommendations, or (3) evidence of new-onset symptoms not attributable to other causes (e.g., flu-like illness) with concurrent laboratory evidence of infection following CDC recommendations. Patients also required evidence of antimicrobial therapy directed at LD (e.g., doxycycline, amoxicillin, cefuroxime, and ceftriaxone). PTLD symptoms, including fatigue, musculoskeletal pain, or neurocognitive complaints, must have begun within two years of LD diagnosis. Self-reported inclusion criteria, assessing severity of symptoms, required that one or more symptoms limit daily function at least half of the time when present. Patients were excluded for several pre-existing conditions, including autoimmune disease (e.g., RA before LD), mood disorders (e.g., major depression and bipolar disease), fibromyalgia, chronic fatigue syndrome, substance abuse, and unexplained chronic pain preceding LD. 

**Figure 1:**
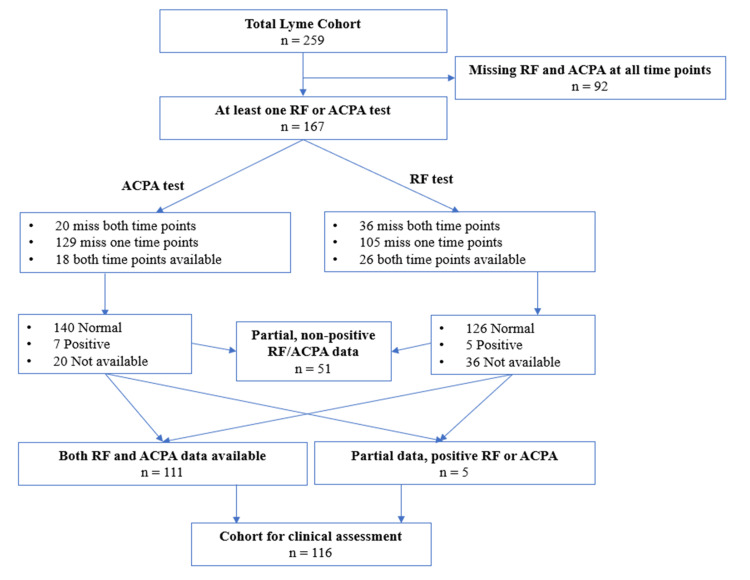
Flowchart of patients included in the study RF, rheumatoid factor; ACPA, anti-citrullinated peptide antibodies

We then examined the availability of RF and ACPA tests in patients’ medical records drawn as close as possible to two points: (1) at the time of initial LD diagnosis and (2) at the time of first evaluation for PTLD in the LDRC. The test results from these two time points were consolidated into a single result for RF and ACPA, respectively. In cases where results were available at both time points, priority was given to the results from the second time point. If data from one time point was missing, the results from the available time point were utilized. We then combined the RF and ACPA test results by assigning the combined result as positive if either RF or ACPA was positive. A normal test combined with a missing test result was assigned as missing (Figure 1). RF and ACPA testing were completed as clinically indicated, rather than part of a standardized process. As such, these serologies were tested at different laboratories, each employing its own validated assays. 

Symptom burden was evaluated by generating a total score representing the sum of the 36 items (range, 0-108) included in the post-Lyme questionnaire of symptoms (PLQS) and by generating neurologic and musculoskeletal domain scores by calculating the sum of specific symptoms for each [2]. Lower scores represent lower symptom reporting, whereas higher scores represent higher symptom reporting. For patients with positive RF and/or ACPA, follow-up chart review was pursued to assess for diagnosis of RA after initial presentation to the LDRC.

Data from normally distributed continuous variables, as assessed by the Shapiro-Wilk test, are presented as mean ± standard deviation (range), and data from continuous variables are not normally distributed as median [25th percentile, 75th percentile] (range). Data from categorical variables are presented as count (%). Comparison between groups was conducted using Fisher’s exact tests for categorical variables and t-tests or Wilcoxon rank sum tests for continuous variables as appropriate. A p-value of <0.05 was deemed signiﬁcant. All statistical analyses were performed using R 4.3.1 [8].

## Results

There were 259 patients meeting the strict definition of PTLD included in this study, and we estimate that approximately 16% of patients referred to the LDRC were eligible for and chose to enroll in this study. Ninety-two were excluded because of missing RF and ACPA data, leaving 64.4% (n = 167) with available RF and/or ACPA tests conducted. Of the 167 patients, RF status was documented at least once for 78.4% (131 of 167), and ACPA status was available at least once for 88.0% (147 of 167). Serial RF testing was available for 19.8% (26 of 131), and serial ACPA was available for 12.2% (18 of 147). 

Complete RF and ACPA data were available for 116 patients, defined as having both RF and ACPA testing or having at least one positive RF or ACPA test result. Among these patients, there was no difference in age or sex based on antibody status (Table 1). For tests completed at the second time point, we analyzed the time from the last day of initial antibiotic treatment for LD to the date of test and found that disease duration was similar between both groups (median: 539 days vs. 532 days, p = 0.767). There was no difference in total symptom burden, nor differences in the musculoskeletal or neurologic symptom domains based on the presence or absence of RF and ACPA antibodies. However, patients with missing RF and ACPA data (n = 92) had fewer musculoskeletal symptoms when compared to patients with complete data (n = 116) (3.0 [2.0, 5.0] vs. 4.0 [3.0, 6.0], p = 0.036).

**Table 1:** Seroprevalence of RF and ACPA in patients with PTLD who had both RF and ACPA testing or had at least one positive RF or ACPA test result (n = 116) Continuous variables are reported as median [interquartile range]. RF, rheumatoid factor; ACPA, anti-citrullinated protein antibodies; PTLD, post-treatment Lyme disease; PLQS, post-Lyme questionnaire of symptoms

Variables	Negative RF and ACPA (n = 104)	Positive RF and/or ACPA (n = 12)	p-value
Age	52.50 [39.75, 60.00]	46.50 [39.00, 58.00]	0.443
Gender (male)	60 (57.7%)	7 (58.3%)	1.000
Duration of illness (years)	1.28 [0.52, 3.30]	1.17 [0.90, 2.62]	0.596
Total PLQS score	23.00 [16.25, 33.00]	27.00 [21.50, 35.75]	0.314
Musculoskeletal score	4.00 [3.00, 6.00]	4.00 [3.50, 5.25]	0.830
Neurological score	10.00 [6.00, 14.00]	12.50 [8.00, 14.75]	0.224
Time from PLQS to RF/ACPA test (days)	0.21 [0.21, 28.46]	22.21 [0.21, 267.50]	0.164
Time from antibiotics to RF/ACPA test (days)	531.79 [160.54, 1292.79]	538.79 [137.04, 1260.95]	0.767

RF was positive in 3.8% (five of 131) of patients, and ACPA was positive in 4.8% (seven of 147). A total of 7.2% (12 of 167) patients were found to have a positive RF or ACPA test. Both RF and ACPA testing were available in only seven of these 12 patients, and none had both antibodies. Serial testing was not available for any of the 12 patients. This included seven male and five female patients, with an average age of 45 ± 15 years. Among those with a positive RF, one patient had a high-titer RF (259 IU/mL), though the other four patients all had titers between 15 and 25 IU/mL (upper limit of normal 14 IU/mL). Among those with a positive ACPA, four patients had low-titer ACPA (30.5 ± 4.2 units), though three patients had titers ≥239 units. While patients with autoimmune disease were excluded at study enrollment, 25% (three of 12) with RF or ACPA were later diagnosed with RA, with all RA diagnoses occurring within two years of LD. Of those with RA, two had low-titer ACPA (26 units and 31 units), and one had high-titer ACPA (>250 units). Of those with high-titer ACPA (≥239 units), only one patient was diagnosed with RA. For those not diagnosed with RA, follow-up was available for more than two years for all but one patient, with an average follow-up of 8.4 ± 5.8 years. 

We then assessed whether RF and ACPA were associated with the initial Lyme presentation (e.g., arthritis, neuroborreliosis, and carditis). There was a trend toward a higher prevalence of RF in patients with an initial diagnosis of neuroborreliosis (18.2% vs. 2.9%, p = 0.072), though there otherwise was no difference in the proportion of patients with RF and/or ACPA based on other initial manifestations (p > 0.58). Interestingly, no patients with initial Lyme arthritis (n = 21) were found to have a positive RF or ACPA in this study.

## Discussion

Our retrospective, observational study is the largest to evaluate the prevalence of RF and ACPA in patients with PTLD. The prevalence of these autoantibodies is similar to what was described in a smaller cohort (n = 85) of patients with PTLD, in which RF was found in 6% of patients [9]. While the proportion of patients with PTLD with a positive RF in both studies is similar to the expected seroprevalence of healthy individuals (occurring in 1-6% of the healthy population), our study is the first to describe ACPA, testing which is strongly associated with development of RA, from patients with PTLD [10-12].

RF positivity has previously been shown to be associated with later manifestations of untreated LD, with studies suggesting that up to 28% (seven of 25) of patients with Lyme arthritis and 70% (seven of 10) with neuroborreliosis made a RF, compared to only 13% (two of 15) with early, EM [13]. These findings were supported by another study showing RF positivity in 20% (four of 20) of patients with Lyme arthritis and 15% (three of 20) of patients with neuroborreliosis [14]. However, longitudinal assessment showed that RF typically became negative after antibiotics [13]. Compared to previous studies, we found a lower proportion of RF in patients with prior Lyme arthritis (0% vs. 20-25%), though this difference likely relates to preceding antimicrobial therapy and longer disease duration in our cohort [14]. However, we found a similar proportion of patients with PTLD after neuroborreliosis had a positive RF (18% vs. 15-70%), despite longer disease duration at the time of evaluation in our study [13,14]. 

While ACPA is highly specific for RA, it is also estimated to be present in about 3-5% of healthy individuals, though few studies have evaluated this in the setting of LD [15,16]. Previous smaller studies were unable to detect ACPA in patients with PTLD [9]. Our study is the first to describe ACPA in a larger PTLD cohort, which was detected in 4.8% (seven of 147) of patients with available data and 2.7% (seven of 259) from all patients with well-defined PTLD, and of those with positive ACPA, nearly half (three of seven) were later diagnosed with RA. The prevalence of RF and ACPA in our PTLD cohort is lower than that seen in patients with post-Lyme RA (7.2% vs. 40.0%, p < 0.001) [3]. 

While patients with autoimmune disease were excluded at enrollment, one limitation is that this study was not designed to identify new autoimmune arthritis, which may develop after the initial clinical visit. Arbuckle et al. described the notion of pre-clinical autoimmune disease, in which autoantibodies may be present years before a patient develops rheumatic disease [17]. While only three of 12 patients with RF or ACPA were diagnosed with RA, it is possible that other patients may develop RA in the future, and longitudinal follow-up would be necessary to determine if there is an increased risk for later development of RA. While a few patients were diagnosed with RA in this study, we interpret this cautiously, given the sample size and incomplete follow-up for all patients. Of those who were not diagnosed with RA, all but one (eight of nine) had medical records available for more than two years after LD diagnosis (8.4 ± 5.8 years). It is important to note that most patients developed RA within two years of LD in our study. This was also observed in the study by Arvikar et al., where RA was typically diagnosed within four months of LD [3]. 

Another limitation is that RF and ACPA testing were abstracted from medical records, not completed in a standardized way as part of a research protocol. Therefore, there are also likely biases about which patients were tested, and our findings may not be generalizable to a broader population of patients with PTLD. It is important to note that 64% of patients (167 of 259) in this study had RF and/or ACPA tested clinically at one or multiple points over the course of their illness, likely to address persistent arthralgia. This is supported by the higher musculoskeletal scores (e.g., more severe symptoms) in patients with RF and/or ACPA testing compared to those without RF and ACPA testing. These limitations emphasize the need for standardized, prospective protocols in the future to assess whether these findings are generalizable to the broader PTLD population. 

RF and ACPA were also tested at different laboratories, each employing their own validated assays, which may also introduce variability in test interpretation, including the potential for false positive testing. While we need to interpret data cautiously in this setting, we feel that these findings are still relevant to the clinician ordering serologies for patients with arthralgia after LD. 

However, we caution that these serologies are not sufficient to determine whether a patient has a new autoimmune arthritis. In the study by Arvikar et al., there was a low proportion of positive RF and ACPA tests, only detected in 20% (six of 30) of all patients with post-Lyme autoimmune arthritis [3]. This highlights that the clinician should not rely solely on serologies when evaluating arthralgia after LD and reinforces the need for more sensitive tools, such as musculoskeletal ultrasound and clinical scoring tools as adjuncts, when evaluating joint pain [18]. This may be particularly important in patients with post-Lyme psoriatic arthritis, where enthesitis may be a key and unrecognized feature [3].

## Conclusions

Using a large cohort of patients with well-defined PTLD, this study found that RF and ACPA testing were frequently utilized clinically, though these autoantibodies were found infrequently in this population. Most of those with positive serologies had low-titer RF or ACPA, and only a small portion were later diagnosed with RA. However, of those with positive ACPA, nearly half went on to develop RA. While RF and ACPA testing had limited yield in the setting of PTLD, those with positive serologies should be monitored closely for the development of an inflammatory arthritis.
